# A New Oleanane Type Saponin from the Aerial Parts of *Nigella sativa* with Anti-Oxidant and Anti-Diabetic Potential

**DOI:** 10.3390/molecules25092171

**Published:** 2020-05-06

**Authors:** Amna Parveen, Muhammad Asim Farooq, Whang Wan Kyunn

**Affiliations:** 1 College of Pharmacy, Gachon University, Hambakmoero, Yeonsu-gu, Incheon 406-799, Korea; 2 Department of Pharmaceutics, School of Pharmacy, China Pharmaceutical University, Nanjing 211198, China; asim@stu.cpu.edu.cn; 3 College of Pharmacy, Chung-Ang University, Seoul 100-031, Korea; whang-wk@cau.ac.kr

**Keywords:** *Nigella sativa*, antioxidant, column chromatography, characterization, isolation, triterpenoid saponin derivatives, flavonoids

## Abstract

Natural product studies explore potential and interesting new compounds to discover innovative drugs. *Nigella sativa* (*N. sativa)* (Ranunculaceae) is traditionally used to treat diabetes. Flavonoids and triterpenoid mostly show anti-diabetic activity. The current study aim to identify new compounds by a systematic study of the anti-oxidant and anti-diabetic activity of aerial parts of *N. sativa* concerning. Phytochemicals were isolated from the methanolic extract of aerial parts of the plant by column chromatography and identified by nuclear magnetic resonance spectroscopy and mass spectroscopy. A new triterpenoid saponin glycoside was isolated along with flavonoids. The anti-diabetic study was carried out by DPPH, ABTS, α-glucosidase, and protein tyrosine phosphatase 1B assays at doses of 12.5 to 250 µM. The isolated phytochemicals were identified as 3-O-(β-d-xylopyranosyl-(1-3)-α-l-rhamnopyrnaosyl-(1-2)-α-l-arabinopyranosyl]-28-O-(α-l-rhamno-pyranosyl-(1-4)-β-d-glucopyranosyl-(1-6)-β-d-glucopyranosyl] hederagenin (**1**), flaccidoside III (**2**), catechol (**3**), quercetin-3-gentiobiosides (**4**), magnoflorine (**5**), nigelflavonoside B (**6**), nigelloside (**7**), quercetin sphorotrioside (**8**), kaempferol-3, 7-diglucoside (**9**), kaempferol 3-O-rutinoside (**10**), rutin (**11**), 3-O-[α-l-rhamnopyranosyl-(1→2)-α-l-arabinopyranpsylhederagenin (**12**), 3β,23,28-trihydroxyolean-12-ene-3-O-α-l-arabinopyranoside(1→4)-a-rhamnopyranosyl,(1→4)-β-d-gluco-pyranoside (**13**), 3-O-α-l-rhamnopyranosyl-(1→2)-α-l-arabinopyranpsyl]-28-O-β-d-gluco-pyranosyl hederagenin (**14**), and α-hederin (**15**). These were isolated and are reported for the first time in this study. Compared **13** was identified as a new compound. Compound **2** was isolated for first time from the genus *Nigella*. Compound **6** was found to be the most active in the DPPH, and ABTS assays and compound **10** was found to be the most active in the α-glucosidase assay, with IC_50_ 32.7 ± 0.1, 95.18 ± 0.9, 214.5 ± 0.0 µΜ, respectively. Compound **12**, at a dose of 125 µΜ, showed anti-diabetic activity in a PTP1B assay with IC_50_ 91.30 ± 2.5 µΜ. In conclusion, the anti-diabetic activity of *N. sativa* is due to its flavonoids and TTSGs. Therefore, our studies suggest that the aerial parts of *N. sativa* are also a valuable and alternate source of valuable phytochemicals that could be used to develop anti-oxidant and anti-diabetic medicines.

## 1. Introduction

Medicinal plants and their derived components have provided potent drugs for modern medicine including vincristine, quinine, morphine, and digoxin to fight against life-threatening diseases such as diabetes, cancer, coronary heart diseases, HIV/Aids, and neurological conditions [1]. Plant-derived molecules are also significant as pharmacological probes, drug prototypes and drug precursors that lead to the discovery of bioactive and innovative compounds or drugs [2,3,4]. Therefore scientists are actively seeking new compounds from natural and traditional resources. *Nigella sativa* (black seeds, *N. sativa*) belongs to the family Ranunculaceae and is emerging as a miracle herb with broad-spectrum pharmacological properties. Its use has a historical and religious background. According to the Islamic point of view narrated in a Hadith “black seed can cure every disease except death [5,6,7]. It is cultivated in many countries, including India, Pakistan, Turkey, Saudi Arabia, and the Middle-Eastern Mediterranean region. It is an annual flowering plant of some 20–90 cm in length. The leaves are finely divided. The segments of the leaf are narrowly linear or threadlike [8,9]. Various biological studies have proved that the seeds contain various pharmacological properties, including anti-diabetic, anti-cancer, anti-hypertensive, anti-inflammatory, bronchodilator, hepatoprotective, nephroprotective, anti-oxidant, gastroprotective, and anti-hypertensive activity.

Currently, diabetes mellitus (DM) is a major and fast-growing disease in communities around the world. Since the year 2011, 346 million people suffer from diabetes, and this prevalence is supposed to double by the year 2030 [10]. Among diabetic patients, ninety percent of these patients were diagnosed with type 2 DM, characterized by chronic high glucose levels linked with carbohydrate, fat, and protein metabolism abnormalities [11,12,13]. According to the American Diabetes Association report, only in the United States about 22.3 million people suffer from diabetes, which represents about 7% of the population [14].

Scientists and researchers are working hard to explore not only the causative factors of diabetes but also to find new molecules to target those factors in order to develop novel therapies. Among the common causative factors of diabetes several, such as auto-oxidation of glucose that results from the free radical formation, receptor tyrosine kinases, including insulin receptor and epidermal growth factor receptor, mediate protein tyrosine phosphatase (PTPs), which are involved in the down-regulation of various cellular signaling transduction mechanisms, and the enzyme glucosidase may be considered as an initial target to screen out potential antioxidant and antidiabetic molecules in order to find innovative molecules via various ways [15,16,17,18].

Considering the importance of this plant, various studies were conducted on its seeds to explore its therapeutic potential. However, there is a need to also explore the pharmacological activities of unstudied parts of this plant, hence the current study was designed to explore the phytochemical composition of the aerial parts of *N. sativa* with regard their potential antidiabetic and anti-oxidant activity.

## 2. Results

### 2.1. Extraction, Isolation, and Identification of Phytochemicals

**Figure 1:**
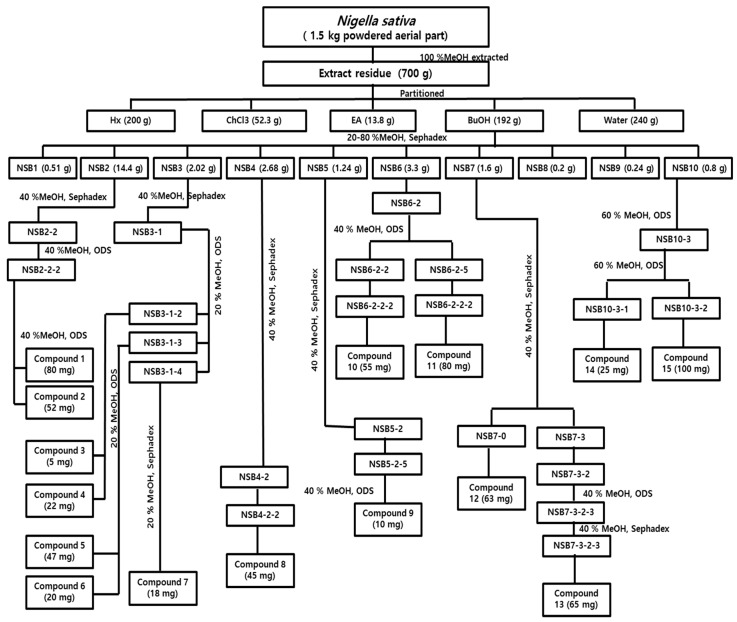
Isolation scheme for *Nigella sativa.*

**Figure 2:**
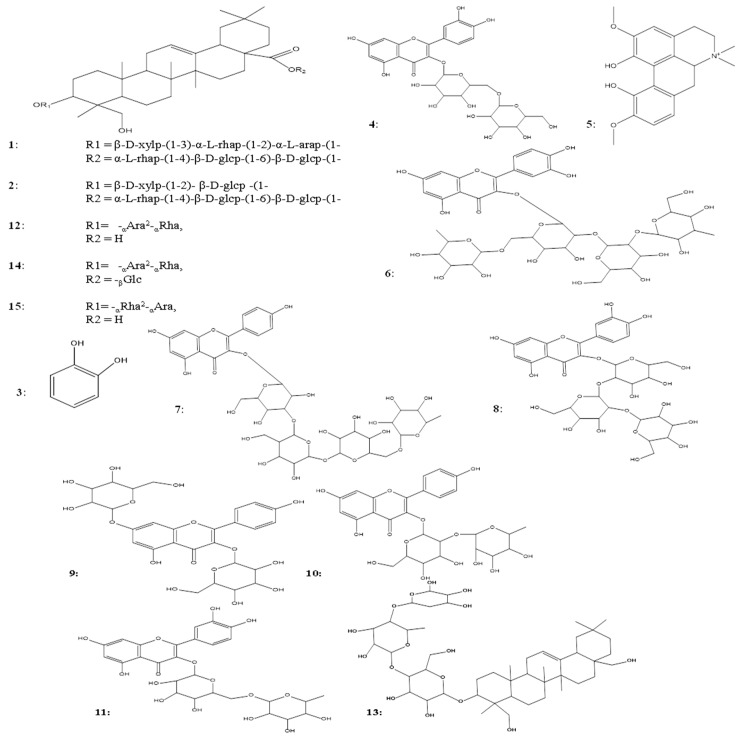
Molecular structures of isolated compounds from *Nigella sativa.* 3-O-[β-d-xylopyranosyl-(1-3)-α-l-rhamnopyrnaosyl-(1-2)-α-l-arabinopyranosyl]-28-O-(α-l-rhamnopyrnaosyl-(1-4)-β-d-gluco-pyranosyl-(1-6)-β-d-glucopyranosyl]hederagenin (**1**), flaccidoside III (**2**), catechol (**3**), quercetin-3-gentiobioside (**4**), magnoflorine (**5**), nigelflavonoside B (**6**), nigelloside (**7**), quercetin sphorotrioside (**8**), kaempferol-3,7-diglucoside (**9**), kaempferol 3-O-rutinoside (**10**), rutin (**11**), 3-O-[α-l-rhamnopyranosyl-(1→2)-α-l-arabinopyranpsyl]hederagenin (**12**), 3β,23,28-trihydroxyolean-12-ene 3-O-α-l-arabinopyranoside (1→4)-a-l-rhamnopyranosyl (1→4)-β-d-glucopyranoside (**13**), 3-O-[α-l-rhamnopyranosyl-(1→2)-α-l-arabinopyranpsyl]-28-O-β-d-glucopyranosylhederagenin (**14**), α-hederin (**15**).

The result showed that all fractions, except the hexane one, have significant DPPH assay activity. Many studies have been done for the isolation of compounds from chloroform fractions of seed extracts, and in this work the chloroform fraction showed the highest activity at the dose of 200 µg with the activity iof other fractions decreasing in the order water > *n*-BuOH > ethyl acetate. The water fraction is hygroscopic, so the butanol fraction was chosen for further isolation. From the butanol fraction, 11 subfractions were obtained by using open column chromatography, and after repeated column chromatography, fifteen compounds were obtained, among which seven compounds (**6**–**11**) were flavonoids, six compounds (**1,2,12**–**15**) were triterpenoid glycosides, one (**5**) was an alkaloid, and one (**3**) was a benzenediol which were further identified by NMR spectroscopy and mass spectroscopy. To the best of our knowledge nobody has reported the isolation of compounds from the aerial parts of *N. sativa* so we have isolated and reported them for the first time. One more interesting finding of our work was the isolation and characterization of the new compound **13**. The isolation scheme is presented in Figure 1. The structures of the isolated compounds are shown in Figure 2.

*3-O-[β-d-xylopyranosyl-(1→3)-α-l-rhamnopyrnaosyl-(1→2)-α-l-arabinopyranosyl]-28-O-[α-l-rhamno-pyranosyl-(1→4)-β-d-glucopyranosyl-(1-6)-β-d-glucopyranosyl]hederagenin* (**1**): ^1^H-NMR (CD_3_OD) δ ppm: 5.35 (1H, d, *J* = 8.22, H-1′′′′′), 5.31 (1H, d, *J* = 8.16, H-1′′′′′′), δ 5.25 (1H, t, H-12), 5.22 (1H, s, H-1′′), 5.22 (1H, s, H-1′′′′), 4.55 (1H, d, *J* = 5.28 H-1′′′), 4.51 (1H, d, *J* = 5.64, H-1′), 3.1-4.1 (sugar moieties overlapped), 0.70 (3H, s, H-24 ) 0.79 (3H, s, H-26), 0.90 (3H, s, H-29), 0.94 (3H, s, H-30), 0.97 (3H, s, H-25), 1.16 (s, 3H each, H-27) 1.23 (m, 6H, 2CH3, H-6′′ and H-6′′′′). ^13^C-NMR (CD_3_OD) δ ppm: 12.41 (C-24), 15.13 (C-25), 16.46 (C-6′′′′), 16.59 (C-6′′), 17.43 (C-6), 22.63 (C-30), 22.67 (C-16), 23.15(C-11), 24.93(C-27), 25.01(C-2), 27.52(C-15), 30.12(C-20), 31.01(C-7 and 22), 31.94( C-29). 33.48 (C-21), 36.23 (C-10), 38.2 (C-1), 38.38(C-8), 41.12(C-18), 41.61(C-14), 42.58(C-4), 45.82(C-17), 46.3 (C-19), 46.65 (C-5), 48.1 (C-9), 63.18 (C-23), 76.58 (C-3), 94.35 (C-1′′′′′), 101.1(C-1′′′′),101.3 (C-1′′)102.95 (C-1′′′), 103.22 (H-1′′′′′′), 105.15 (C-1′), 122.39 (C-12), 143.50 (C-13), 176.68 (C-28) ), and other signals similar to literature data [19].

*Flaccidoside III* (**2**) (detected for first time in the genus *Nigella*): ^1^H-NMR (CD_3_OD) δ ppm: 5.33 (1H, d, *J* = 7.38, H-1′′′′′), 5.25 (1H, t, H-12), 5.23 (1H, s, H-1′′), 5.22 (1H, s, H-1′′′′), 4.51 (1H, d, *J* = 5.58, H-1′), δ 4.49 (1H, d, *J* = 7.38, H-1′′′′′), δ 4.41 (1H, d, *J* = 7.38, H-1′′′), 3.3-4.1 (sugar moieties overlapped), 0.70 (3H, s, H-24 ) 0.80 (3H, s, H-26), 0.91 (3H, s, H-29), 0.94 (3H, s, H-30), 0.98 (3H, s, H-25), 1.16 (s, 3H each, H-27) 1.23 (m, 6H, 2CH3, H-6′′ and H-6′′′′). ^13^C-NMR (CD_3_OD) δ ppm: 176.74 (C-28), 143.51 (C-13), 122.68 (C-12), 105.15 (C-1′), 103.3 (C-1′′′), 101.52(C-1′′), 99.93 (C-1′′′′), 94.35 (C-1′′′′′), 76.58 (C-3), 65.60 (C-6′′′), 64.10 (C-6′′′′), 63.18 (C-23), 60.47 (C-6′′′′′), 48.1 (C-9), 46.65 (C-5), 46.3 (C-19), 45.82 (C-17), 42.58 (C-4), 41.61(C-14), 41.11 (C-18), 39.27 (C-8), 38.33 (C-1), 36.24 (C-10), 33.52 (C-21), 32.09 (C-29), 31.87 (C-7 and 22), 30.14 (C-20), 27.53 (C-15), 25.20 (C-2), 24.97(C-27), 23.17(C-11), 22.64 (C-16), 22.64 (C-30), 17.44 (C-6), 16.62 (C-6′′), 15.17 (C-25), 12.44 (C-24), and other signals similar to literature data [19].

*Catechol* (**3**): ^1^H-NMR (CD_3_OD) δ ppm: 8.18(s, 2H, H-3 and 6), 8.11 (s, 2H, H-4 and 5). ^13^C-NMR (CD_3_OD) δ ppm: 152.25 (C-1 and 2), 139.79 (C-3 and 6) [20].

*Quercetin-3-gentiobioside* (**4**): ^1^H-NMR (CD_3_OD) δ ppm: 7.77 (1H, dd, *J* = 2.16, 8.58, H-6′), 7.65 (1H, d, *J* = 2.22, H-2′), 7.35 (1H, d, *J* = 8.64, H-5′), 6.3 (1H, s, H-8), 6.1 (1H, s, H-6), 5.03 (1H, d, *J* = 7.26, H-1′′), 4.72 (1H, d, *J* = 7.74, H-1′′′) 3.2-3.9 (disaccharide moieties overlapped). ^13^C-NMR (CD_3_OD) δ ppm: 159.0 (C-2), 135.0 (C-3), 179.6 (C-4), 163.0 (C-5), 99.8 (C-6), 166.0 (C-7), 94.7 (C-8), 158.4 (C-9), 105.1 (C-10), 122.8 (C-1′), 116.2 (C-2′), 146.1 (C-3′), 149.8 (C-4′), 117.4 (C-5′), 123.1 (C-6′), 104.5 (C-1′′), 75.6 (C-2′′), 78.2 (C-3′′), 71.2 (C-4′′), 77.7 (C-5′′), 66.2 (C-6′′) and other signals similar to literature values [21].

*Magnoflorine* (**5**): ^1^H-NMR (CD_3_OD) δ ppm: 6.61 (1H, d, *J* = 7.86, H-8), 6.38 (1H, d, *J* = 7.8, H-9), 6.34 (1H, s, H-3), 3.80 (3H, s, OMe-2), 3.71 (3H, s, OMe-10), 3.60 (2H, m, H-5), 3.14 (3H, s, N-CH3), 3.02 (2H, m, H-4), 2.65 (3H, s, N-CH_3_). ^13^C-NMR (CD_3_OD) δ ppm: 151.51(C-2), 150.2(C-10), 149.34 (C-11), 148.40 (C-1), 124.6 (C-7a), 122 (C-8), 119.59 (C-3a), 115.5 (11-a), 114.3 (1-a), 109.13 (C-9), 107.94 (C-3), 69.44 (C-6a), 60.74 (C-5), 54.89 (OMe-C10), 54.59 (OMe C11), 52.43, 42.122 (N ^±^ Me), 30.15 (C-7), 23.18 (C-4) and other signals as reported in the literature [22].

*Nigelflavonoside B* (**6**): ^1^H-NMR (CD_3_OD) δ ppm: 7.7 (1H, d, *J* = 2.16, H-6′), 7.55 (1H, dd, *J* = 2.16, 8.46, H-2′), 6.91 (1H, d, *J* = 8.46, H-3′), 6.36 (1H, s, H-8), 6.17 (1H, s, H-6), 5.24 (1H, d, *J* = 7.26, H-1′′), 4.73 (1H, d, *J* = 7.74, H-1′′′), 4.67 (1H, d, *J* = 7.68, H-1′′′′), 4.50 (1H, s, H-1′′′′′), 3.2-3.9 (disaccharide moieties overlapped), 1.10 (3H, d, H-6′′′′′). ^13^C-NMR (CD_3_OD) δ ppm: 177.9 (C-4), 164.38 (C-7), 161.6 (C-5), 157.03 (C-9 and 2), 148.3 (C-4′), 144.30 (C-3′), 133.4 (C-3), 121.84 (C-1′ and 6′), 116.43 (C-2′), 114.84 (C-5′), 105.01 (C-10), 104.25 (C-1′′′′), 103.61 (C-1′′′), 100.67 (1′′′′′), 99.59 (C-6), 98.47 (C-1′′), 83.83 (C-2′′), 82.27 (C-2′′′), 77.39 (C-5′′′′), 76.19 (C-3′′′′), 75.93 (C-3′′), 75.65 (C-5′′), 74.91 (5′′′), 73.63 (C-3′′′), 72.49 (C-6′′), 70.74 (C-4′′′′′), 70.64 (C-3′′′′′), 69.59 (C-2′′′′′), 69.16 (C-4′′), 60.82 (C-6′′′′), 60.43 (C-6′′′), 16.45 (C-6′′′′′).

*Nigelloside* (**7**): ^1^H-NMR (CD_3_OD) δ ppm: 8.05 (2H, d, *J* = 8.82, H-2’ and 6′), 6.94 (2H, d, *J* = 8.82, H-3′ and 5′), 6.36 (1H, s, H-8), 6.17 (1H, s, H-6), 5.39 (1H, d, *J* = 7.5, H-1′′), 4.73 (1H, d, *J* = 7.68, H-1′′′), 4.67 (1H, d, *J* = 7.68, H-1′′′′), 4.50 (1H, s, H-1′′′′′), 3.2-3.9 (disaccharide moieties overlapped), 1.13 (3H, d, H-6′′′′′). ^13^C-NMR (CD_3_OD) δ ppm: 178.1 (C-4), 164.43 (C-7), 161.63 (C-5), 159.96 (C-4′), 157.06 (C-2,9), 133.2 (C-3), 131.84 (C-2′ and 6′), 121.43 (C-1′), 114.89 (C-3′, 5′), 104.33 (C-10), 103.39 (C-1′′), 100.72 (1′′′′), 99.40 (C-1′′′′′), 98.47 (C-6), 83.83 (C-2′′), 82.27 (C-2′′′), 77.39 (C- 5′′′′), 76.19 (3′′′′), 75.93 (C-3′′), 75.65 (C-5′′), 74.91 (5′′′), 73.59( C-3′′′), 72.49 (C-6′′), 70.74 (C-4′′′′′), 70.64 (C-3′′′′′), 69.59 (C-2′′′′′), 69.29 (C-4′′), 68.27 (C-4′′′′), 66.57 (C-6′′), 60.90 (C-6′′′′), 60.43 (C-6′′′), 16.45 (C-6′′′′′). Other signals are similar to literature data [23]. 

*Quercetin sphorotrioside* (**8**): ^1^H-NMR (CD_3_OD) δ 7.75 (1H, d, *J* = 2.16, H-6′), 7.55 (1H, dd, *J* = 2.16, 8.46, H-2′), 6.91 (1H, d, *J* = 8.46, H-3′), 6.35 (1H, s, H-8), 6.16 (1H, s, H-6), 5.29 (1H, d, *J* = 7.14, H-1′′), 4.73 (1H, d, *J* = 7.68, H-1′′′), 4.67 (1H, d, *J* = 7.38, H-1′′′′), 3.2-3.9 (disaccharide moieties overlapped), 1.10 (3H, d, H-6′′′′′). ^13^C-NMR (CD_3_OD) δ ppm: 177.9 (C-4), 164.38 (C-7), 161.67 (C-5), 157.03 (C-9 and 2), 148.3 (C-4′), 144.30 (C-3′), 133.4 (C-3), 121.84 (C-1′ and 6′), 116.36 (C-2′), 114.83 (C-5′), 105.07 (C-1′′′′), 104.35 (C-1′′′), 103.659 (C-10), 99.61 (C-6), 98.47 (C-1′′), 83.83 (C-2′′), 82.27 (C-2′′′), 77.39 (C- 5′′′), 76.95 (C-5′′′′)76.19 (C-5′′), 75.93 (C-3′′), 75.65 (C-5’’), 74.91 (3’’),74.78 (C-3’’’’), 73.63( C-2’’’’), 69.59 (C-4′′), 68.83 (C-4′′′′),68.26 (C-6′′) 60.93 (C-6′′′′), 60.39 (C-6′′′) and other signals similar to literature values [24].

*Kaempferol-3, 7-diglucoside* (**9**): ^1^H-NMR (CD_3_OD) δ ppm :8.08 (2H, d, *J* = 8.88, H-2′ and 6′), 6.90 (2H, d, *J* = 8.76, H-3′ and 5′), 6.38 (1H, s, H-8), 6.19 (1H, s, H-6), 5.33 (1H, d, *J* = 7.62, H-1′′), 4.74 (1H, d, *J* = 7.68, H-1′′′), 3.3-3.8 (sugar moieties). ^13^C-NMR (CD_3_OD) δ ppm: 178.38 (C-4), 164.82 (C-7), 160.14 (C-5), 157.22 (C-4′), 157.08 (C-2,9), 133.48 (C-3), 130.94 (C-2′ and 6′), 121.27 (C-1′), 114.84 (C-3′, 5′), 104.22 (C-10), 103.39 (C-1′′′), 100.08 (1′′), 98.50 (C-6), 93.33 (C-8), 77.4 (C-3′′,5′′,3′′′, 5′′′) 74.06 and 73.42 (C-2′′′ and2′′), 69.89 (C-4′′), 69.7(C-4′′′), 61.1 (C-6′′, 6′′′) and other signals similar to literature data [25].

*Kaempferol 3-O-rutinoside* (**10**): ^1^H-NMR (CD_3_OD) δ 8.05 (2H, d, *J* = 8.76, H-2′ and 6′), 6.87 (2H, d, *J* = 8.76, H-3′ and 5′), 6.38 (1H, s, H-8), 6.19 (1H, s, H-6), 5.12 (1H, d, *J* = 7.44, H-1′′), 4.51 (1H, s, H-1′′′), 3.3-3.8 (sugar moieties), 1.12 (3H, d, *J* = 6.20, H-6′′′). ^13^ C-NMR (CD_3_OD) δ ppm: 177.94 (C-4), 164.64 (C-7), 161.53 (C-5), 160.04 (C-4′), 157.96 (C-9), 157.08 (C-2), 134.10 (C-3), 130.94 (C-2′ and 6′), 121.30 (C-1′), 114.71 (C-3′, 5′), 104.22 (C-10), 103.22 (C-1′′), 101.08 (1′′′), 98.50 (C-6), 93.33 (C-8), 76.72 (C-3′′), 75.77 (C-5′′), 74.31 (C-2′′), 72.48 (C-4′′), 70.068(C-4′′′), 70.61 (C-2′′′), 69.99 (C-3′′′), 68.30 (C-5′′′), 67.17 (C-6′′), 16.50 (C-6′′′) and other signals similar to literature data [26].

*Rutin* (**11**): ^1^H-NMR (DMSO-d_6_) δ ppm: 7.5 (2H, s, H-2′ and 6′), 6.8 (1H, s, H-5′), 6.36 (1H, s, H-8), 6.17 (1H, s, H-6), 5.31 (1H, s, H-1′′), 4.36 (1H, s, H-1′′′), 3.2-3.9 (disaccharide moieties overlapped), 0.97 (3H, H-6′′′′′). ^13^C-NMR (DMSO-d_6_) δ ppm: 177.67 (C-4), 164.86 (C-7), 161.63 (C-5), 156.96 (C-9 and 2), 148.95 (C-4′), 145.22 (C-3′), 133.69 (C-3), 122.03 (C-1′), 121.53 (c-6′), 116.43 (C-5′), 114.84 (C-2′), 104.21 (C-10), 101.72 (1′′′), 101.17 (C-1′′′), 99.29 (C-6), 94.15 (C-8),76.90 (C-3′′), 76.32 (C-5′′), 74.50 (C-2′′), 72.28 (C-4′′′), 70.99 (C-4′′, 2′′′), 70.41 (C-3′′′), 68.65 (C-5′′′), 67.39 (C-6′′), 18.17 (C-6′′′) and other signals similar to those reported in the literature [27].

*3-O-[α-l-Rhamnopyranosyl-(1→2)-α-l-arabinopyranpsyl]hederagenin* (**12**): ^1^H-NMR (CD_3_OD) δ ppm: 5.15 (1H, t, H-12), 5.14 (1H, s, H-1′′), 4.55 (1H, d, *J* = 5.16, H-1′′′), 3.2-3.9 (sugar moieties), 0.69 (3H, s, H-24 ) 0.82 (3H, s, H-26), 0.93 (3H, s, H-29), 0.96 (3H, s, H-30), 0.99 (3H, s, H-25), 1.18 (s, 3H each, H-27) 1.23 (3H, CH3, H-6′′′). ^13^C-NMR (CD_3_OD) δ ppm: 178.88 (C-28), 142.93 (C-13), 122.56 (C-12), 102.87 (C-1′′), 100.47 (C-1′), 80.84 (C-3),75.25 (C-2′′) 72.51 (C-3′), 72.23(C-4′), 71.14 (C-3′), 70.73 (C-2′), 70.60 (C-4′′), 68.75 (C-5′, and 5′′), 63.18 (C-23), 52.19 (C-9), 47.45 (C-5), 47.59 (C-19), 47.51 (C-17), 42.77 (C-4), 42.52 (C-14), 41.84 (C-18), 39.29 (C-8), 38.23 (C-1), 36.18 (C-10), 32.18 (C-21), 31.87 ( C-29), 30.76 (C-7 and 22), 26.90 (C-20), 25.58 (C-15), 25.09(C-2), 23.79 (C-27), 23.13 (C-11 and 30), 17.39 (C-16), 16.55 (C-6), 16.38 (C-6′), 15.58 (C-26), 14.94 (C-25), 12.32 (C-24).

*3β,23,28-Trihydroxyolean-12-ene-3-O-α-l-arabinopyranosyl-(1→4)-α-l-rhamnopyranosyl-(1→4)-β-d-gluco-pyranoside* (**13**) (new compound): ^1^H-NMR (C_5_D_5_N) δ ppm: 5.22 (1H, s, H-12), 3.2-3.9 (sugar moieties), 3.82 (1H, H-3), 3.50 (2H, H-23), 3.34 (2H, H-28), 0.70 (3H, s, H-24 ) 0.87 (3H, s, H-26), 0.92 (3H, s, H-29), 0.97 (6H, H-25 and 30), 1.07 (3H, s, 27), 1.26 (3H, d, *J* = 6.18, H-6′′).^13^C-NMR (C_5_D_5_N) δ ppm: 144.70 (C-13), 121.70 (C-12), 105.08 (C-1′), 103.21 (C-1′′′), 99.99 (C-1′′), 80.99 (C-3′), 80.68 (C-3 and 3′′′), 76.20 (C-5′), 74.79 (C-4′), 73.81 (C-2′′), 72.72 (C-4′′), 71.36 (C-3′′), 70.25 (C-2′′′), 69.66 (C-2′), 68.54 (C-5′′), 68.20 (C-5′′′), 65.59 (C-6′), 63.24 (C-23), 52.17 (C-17), 46.78 (C-5), 46.12 (C-19), 42.58 (C-4), 42.17 (C-14), 42.16 (C-18), 41.29 (C-1 and 21), 39.19 (C-8), 38.30 (C-5′′′), 36.26 (C-10), 32.41 ( C-29), 32.12(C-7), 32.12 (C-22 and 7), 30.65 (C-20), 25.20 (C-15), 27.36 (C-2), 25.27 (C-27), 23.98 (C-30 and 11), 23.8 (C-16), 17.50 (C-6), 16.80 (C-26), 16.66 (C-6′′), 15.12 (C-25), 12.46 (C-24).

*3-O-[α-l-Rhamnopyranosyl-(1→2)-α-l-arabinopyranpsyl]-28-O-β-d-glucopyranosylhederagenin* (**14**): ^1^H-NMR (C_5_D_5_N) δ ppm: 6.33 (1H, s, H-12), 5.35 (1H, d, *J* = 7.38 H-1′′′), 5.09 (1H, d, *J* = 6.42, H-1′′), 4.92 (1H, s, H-1′) 3.2-3.9 (sugar moieties), 0.93 (3H, s, H-24 ) 0.96 (3H, s, H-26), 1.02 (3H, s, H-29), 1.15 (3H, s, 27), 1.26 (6H, s, H-30 and25), 1.58 (3H, CH3, H-6′′′).^13^C-NMR (C_5_D_5_N) δ ppm: 178.88 (C-28), 142.93 (C-13), 122.56 (C-12), 106.09 (C-1′′), 103.24 (C-1′), 99.92 (C-1′′′), 81.61 (C-3), 79.72 (C-3′′′), 79.70 (C-5′′′), 76.91 (C-2′′) 74.8 (C-3′′′) 74.18 (C-4′ and2′′′), 73.84 (C-3′), 72.4 (C-2′), 71.52 (C-3′′′), 69.7 (C-4′′), 68.28 (C-5′), (C-4′′), 65.94 (C-5′′), 64.74 (C-23), 62.63 (C-6′′′), 52.19 (C-9), 47.45 (C-5), 47.59 (C-19), 47.51 (C-17), 42.77 (C-4), 42.52 (C-14), 40.84 (C-18), 39.62 (C-8), 38.30 (C-1), 37.63 (C-10), 32.00 (C-21), 31.49 (C-29), 29.65 (C-7), 28.56 (C-22), 26.90 (C-20), 25.58 (C-15), 24. 74 (C-2), 23.79 (C-27), 22.65 (C-11 and 30), 17.39 (C-16), 16.55 (C-6), 16.38 (C-6′), 15.58 (C-26), 14.94 (C-25), 12.32 (C-24) and other signals are similar according to literature.

*α-Hederin* (**15**): ^1^H-NMR (C_5_D_5_N) δ ppm: 6.52 (1H, s, H-12), 5.71 (1H, s, H-1′′), 5.41 (1H, d, *J* = 6.12, H-1′′), 3.2-3.9 (sugar moieties), 1.21 (3H, s, H-24 ) 1.24 (3H, s, H-26), 1.30 (3H, s, H-29), 1.36 (3H, s, 27), 1.52 (6H, s, H-30 and25), 1.83 (3H, CH3, H-6′′).^13^ C-NMR (C_5_D_5_N) δ ppm: 180.1 (C-28), 145.46 (C-13), 122.15 (C-12), 104.42 (C-1′), 101.24 (C-1′′), 81.61 (C-3), 75.89 (C-2′), 74.75 (C-3′), 74.17(C-4′′), 72.63 (C-3′′), 72.41 (C-2′′), 69.77 (C-5′), 69.40 (C-5′′), 65.77 (C-6′), 64.11 (C-23), 48.28 (C-9), 47.81 (C-4), 46.94 (C-17), 43.59 (C-19 and 5), 42.28 (C-14), 41.07 (C-18), 39.80 (C-8), 39.06 (C-1), 36.99 (C-10), 34.52 (C-21), 33.44 (C-29), 32.96 (C-7 and 22), 31.10 (C-20), 28.59 (C-15), 26.36 (C-27), 26.30 (C-2), 24.03 (C-30), 23.92 (C-11 and 16), 18.62 (C-6), 17.67 (C-26), 16.18 (C-25), 14.07 (C-24) and other signals similar to data recorded in the literature [28].

### 2.2. Structure Elucidation of 3β,23,28-trihydroxyolean-12-ene 3-O-α-l-arabinopyranoside (1→4)-α-l-rhamnopyranosyl (1→4)-β-d-glucopyranoside Compound (13)

Compound **13** was isolated as off-white crystals and its molecular formula was established as C_47_H_78_O_16_ on the basis of a combination of the peaks at *m/z* 897 (M−H)^−^ (calcd 898.53) in the FABMS (negative ion mode) and the 13C-NMR spectrum. Other important ions were observed at *m/z* 765, [M-H-132 (xylose)]^−^, *m/z* 619, [M-H-132 (xylose)–146 (rhamnose)]^−^, *m/z* 439 [(765-H-146-162 (rhamnose and glucose)]^−^, *m/z* 325 (rhamnose and glucose). The 1D NMR spectra (^1^H, ^13^C and DEPT) of compound **1** displayed the presence of seven methyls, one double bond (olefin proton). All signals observed in the upfield (0.6–2 ppm) and midfield (2.8–5.3 ppm) region of ^1^H-NMR spectrum, together with anomeric signals corresponding to three monosaccharide residues in the HSQC spectrum, suggested the compound was a triterpene glycoside. From a careful analysis of 1D, 2D NMR data (COSY, HMBC, and HSQC), the aglycon part was revealed to be a characteristic triterpene having seven methyl singlets at δ 0.70, 0.97, 0.87, 1.07, 0.92, 0.97, and 1.26) an olefin proton together with one anomeric proton of a rhamnose sugar the at δ 5.22 (2H, s, H-1′′) along with two more anomeric protons at δ 4.50 (1H, d, *J* = 7.38 H-1′) and δ 4.53 (1H, d, *J* = 5.16 H-1′′′) indicating the presence of three sugars: glucose, rhamnose and arabinose. The methyl proton of the rhamnose unit resonated at δ 1.26 (3H, d, *J* = 6.18, H-6”). The two -CH_2_OH groups resonated at δ 3.50 (2H, H-23) and 3.34 (2H, H-28). The ^13^C-NMR data showed 47 carbons, of which 30 were ascribed to the aglycone.

A DEPT experiment was used to resolve the 30 carbon resonances of the aglycon into six methyls, ten methylenes, four methines and seven quaternary carbons. The ^13^C-NMR spectrum showed the resonance for five methyl groups δc [12.46, 15.12, 23.98, 25.27, and 32.41], an olefin bond [δc 121.70 and 144.70], an oxygenated methane (δc 80.68) and two -CH_2_OH groups (δc 63.24 and 64.00) related to the aglycone moiety. The aglycone unit of the molecule is identical to that of glomeruloside B. It is also closely related to erythrodiol, a known molecule, based on a survey of the literature. The difference is the presence of another -CH_2_OH at the C-23 position. The remaining seventeen carbon resonances, including the anomeric protons δc [105.08 (C-1′), 99.99 (C-1′′) and 103.21 (C-1′′′) were assigned to three sugar moieties (glucopyranose, rhamnopyranse, and xylopyranose). The long-range HMBC correlations among H-1′/ C-3, H-′′/C-4′ H-1′′′/ C-4′′, revealed the position of glucose at C-3, next is rhamnose at C-4′ and then xylose at C-4′′. Acid hydrolysis showed the presence of L-arabinose, L-rhamnose, and D-glucose as sugar components. The compound’s NMR data also match that of the other oleanane-type saponin glomeruloside B, but the sugar attachment is different [29].

### 2.3. Fingerprint Analysis by HPLC–UV

**Figure 3:**
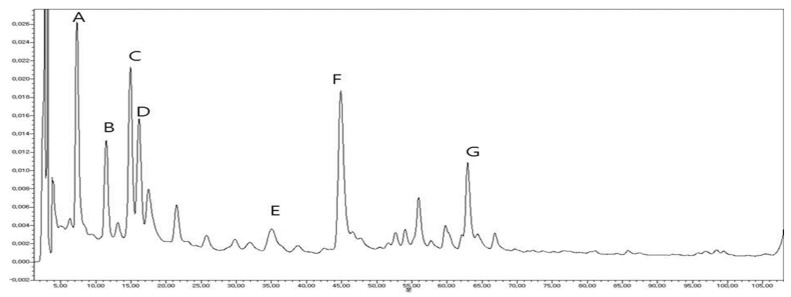
HPLC chromatogram of *Nigella sativa* 70% ethanolic extract. Quercetin-3-gentiobioside (**A**), nigelflavonoside B (**B**), quercetin sphorotrioside (**C**), nigelloside (**D**), kaempferol-3,7-diglucoside (**E**), rutin (**F**), and kaempferol 3-O-rutinoside (**G**).

The HPLC chromatogram determined for *Nigella sativa* under the experimental conditions is shown in Figure 3. Seven peaks identified as quercetin-3-gentiobioside, nigelflavanoside B, quercetin sphorotrioside, nigelloside, rutin, kaemferol-3,7-diglucoside, and kaempferol-3-O-rutinoside were detected. These results strongly suggested that flavonoids contributed to anti-diabetic activity.

### 2.4. Bioassays

#### 2.4.1. Antioxidant Assays

##### DPPH Assay

The DPPH activity of the compounds isolated from *Nigella sativa* is listed in Table 1. Among all the isolated compounds, compounds **5**, **6**, **8**, and **11** showed a significant inhibition of DPPH in a dose-dependent manner with IC_50_ values of 71.0 ± 0.5 μM, 32.7 ± 0.12 μM, 35.5 ± 0.5 μM, and 39.6 ± 0.5 μM, respectively, in comparison with the positive control, ascorbic acid, with IC_50_ 51.7 ± 0.1 μM. 

##### ABTS Assay

The ABTS activity of the compounds isolated from *Nigella sativa* is also listed in Table 1. Among all the isolated compounds, compounds **5**, **6**, **8**, and **11** showed a significant inhibition of ABTS radical in a dose-dependent way with IC_50_ values of 125.8 ± 0.5 μM, 106.0 ± 0.1 μM, 113.5 ± 0.5 μM, and 122.0 ± 0.5 μM, respectively, in comparison with the positive controls, ascorbic acid, and Trolox, with IC_50_ 82.9 ± 2 and 84.3 ± 1.5 μM, respectively.

#### 2.4.2. Anti-Diabetic Assays

##### α-Glucosidase Assay

The α-glucosidase inhibitory activity of the compounds isolated from *Nigella sativa* is presented in Table 1. The results showed that most of the compounds (**1**, **2**, **4**, **5**, **6**, **7**, **8**, **9**, **10**, and **11**) showed activity against α-glucosidase with IC_50_ values of 256.7 ± 3.7 μM, 254.2 ± 4.51 μM, 335.3 ± 0.2 μM, 257.8 ± 0.8 μM, 276.2 ± 2.1 μM, 274.1 ± 0.32 μM, 360.0 ± 0.3 μM, 214.5 0.09 μM, 331.9 ± 1.6 μM, respectively in comparison with the standard drug, acarbose, with IC_50_ 127.9 ± 2.0 μM.

##### PTP1B Assay

In the determination of PTP1B inhibitory activity of isolated compounds of Nigella Sativa are mentioned in Table 1. The results showed that only compound **12** showed significant activity with IC_50_ 91.3 ± 2.5 μM in comparison with the standard drug, Ursolic acid, with IC_50_ 0.8 ± 1.4 μM.

## 3. Discussion

Plants parts such as rhizomes, seeds, stems and leaves are composed of countless chemical constituents that present biological and pharmacological activity against various disease conditions [2,3,4]. In the current study the antidiabetic and anti-oxidant activity of the phytochemicals in the butanol fraction of the aerials part of *N. sativa* was explored. After repeated open column chromatography, fifteen compounds were isolated and identified as 3-O-(β-d-xylopyranosyl-(1-3)-α-l-rhamnopyrnaosyl-(1-2)-α-l-arabinopyranosyl]-28-O-(α-l-rhamnopyrnaosyl-(1-4)-β-d-gluco-pyranosyl-(1-6)-β-d-glucopyranosyl]hederagenin (**1**), flaccidoside III (**2**), catechol (**3**), quercetin-3-gentiobioside (**4**), magnoflorine (**5**), nigelflavonoside B (**6**), nigelloside (**7**), quercetin sphorotrioside (**8**), kaempferol-3,7-diglucoside (**9**), kaempferol 3-O-rutinoside (**10**), rutin (**11**), 3-O-[α-l-rhamnopyranosyl-(1→2)-α-l-arabinopyranpsylhederagenin (**12**), 3β,23,28-trihydroxyolean-12-ene-3-O-α-l-arabinopyranoside(1→4)-α-rhamnopyranosyl-(1→4)-β-d-glucopyranoside (**13**), 3-O-α-l-rhamnopyranosyl-(1→2)-α-l-arabinopyranpsyl]-28-O-β-d-glucopyranosylhederagenin (**14**), and α-hederin (**15**). A detailed study reveals that these were isolated and are reported for the first time in this study. Compound **2** was isolated first time from the genus *Nigella* and compound **13** was identified as a new compound.

Protein tyrosine phosphatase 1B (PTP1B) is one of the members of the protein tyrosine phosphatase family. PTP1B is considered to fucntion as a negative regulator of insulin signal transduction. Dephosphorylation of phosphotyrosine residues occurs when activated insulin receptor or insulin receptor substrate-1 (IRS-1) interacts with PTP1B directly. This dephosphorylation results in the downregulation of the insulin effect [30]. This downregulation enhances insulin sensitivity, which indicates that PTP1B plays a significant role in the modulation of insulin sensitivity. In T2DM, increased hyperglycemia stimulates auto-oxidation of glucose that results from free radical formation. The formation of free radicals causes macro- and microvascular dysfunction, and the diabetic patient’s condition becomes worse with an increase in cardiovascular disease incidence [31,32]. Therefore, the use of antioxidants offers not only protective effects in diabetes but also cardiovascular disease. Hyperglycemia can also be controlled by using α-glucosidase inhibitors that delay glucose absorption after a meal. The enzyme glucosidase causes the conversion of carbohydrates into glucose, and ultimately the glucose level increases [33]. Therefore, PTP1B inhibitors, antioxidant, and α-glucosidase inhibitors are considered as potential targets for a diabetic cure and diabetes-related diseases via various pathways.

To determine the antioxidant activity of the isolated compounds, two radical scavenging assays were used, the DPPH assay and ABTS assay. Results from both tests were similar. The results show that compounds **5**, **6**, **8**, **9**, and **11** appear to have more anti-oxidant activity. Compounds **6**, **8**, **9**, and **11** are flavonoid compounds and showed high anti-oxidant activity. In order to determine the α-glucosidase and PTP1B inhibiting activity, two assays were performed: α-glucosidase and PTP1B inhibition assays. Our findings show that all the isolated flavonoids, along with the alkaloid, have strong potential to inhibit the enzyme α-glucosidase, which is the main enzyme involved in the metabolism of carbohydrates to glucose. Compound **12** isolated from *N. sativa* showed significant activity in a dose-dependent manner against PTP1B. Compound **12** is a derivative of hederagenin, which is obtained in needle-like crystal form. Table 1 presents the bioactivity of the isolated compounds against diabetes. 

In conclusion, 15 different compounds with different chemical structures were isolated from the aerial parts of *N. sativa* for the first time. Their mechanism of action to fight against different disease conditions may be different and multi-targeted, as they belong to different classes of phytochemicals. Oxidative stress, glucosidase enzyme, and PTP1B enzyme are possible causes of diabetes. By reducing and inhibiting these factors, diabetes can be treated more effectively. Therefore, our studies suggest that the aerial part of *N. sativa* may be a valuable and alternatative source of phytochemicals that can be used to develop an anti-oxidant and anti-diabetic medicine containing different classes of compounds. However, further research needs to be carried out to explore its signaling pathways in order to develop an innovative and effective for anti-diabetic therapy.

## 4. Materials and Methods

### 4.1. Chemicals and Equipment

Water HPLC-grade (J. T. Baker®, Phillipsburg, PA, USA), HPLC-grade formic acid (DEAJUNG Chemical, Siheung, Gyeonggi, Korea), methanol-d_4_ (Sigma-Aldrich, St. Louis, MO, USA), HPLC-grade phosphoric acid, dimethyl sulfoxide, ascorbic acid, dinitrosalicylic acid, Trolox, acarbose, pyrogallol, sodium chloride, sodium phosphate monobasic, sodium phosphate dibasic, xanthine oxidase, α-glucosidase, α-amylase, *p*-nitrophenyl phosphate, ursolic acid, protein tyrosine phosphatase 1B, *p*-nitrophenyl-α-d-glucopyranoside (Sigma-Aldrich, St. Louis, MO, USA), Balance; Mettler Toledo ML 204 (Greifensee, Switzerland), Vortex mixer; maxi mix 2 (Thermo Scientific, Selangor, Malaysia), UV/Vis spectrophotometer: Optizen 2120UV (Agilent, Aanta Clare, CA, USA), Mass spectrometer; JMS-700 (JEOL, Japan); fast atom bombardment negative mode, NMR: ^1^H-NMR (600 MHz) and ^13^C-NMR (151 MHz) Gemini 2000 (Varian, Palo Alto, CA, USA). HPLC column; RP-C_18_ Kromasil (250 mm × 4.6 mm, 5 μm).

### 4.2. Collection and Identification of Plant Materials

In August 2019, aerial parts of *N. sativa* were collected from the Faisalabad region of Pakistan, and identified by a plant taxonomist of the Department of Botany, GC University Faisalabad, Pakistan, where a voucher has been submitted. A voucher specimen was also submitted to the Pharmaceutical Resources Botany Laboratory, College of Pharmacy, Chung-Ang University, Korea. The plant material was dried under the shade at temperatures ranging from 21 to 30 °C for 10 to 15 days and ground into a powder.

### 4.3. Extraction, Isolation and Identification of Phytochemicals

About 1.5 kg of the powdered aerial part was extracted with 100% MeOH, and the extract was dried under vacuum on a rotary evaporator. The methanol extract was further partitioned with *n*-hexane, dichloromethane (DCM), ethyl acetate (EA), normal butyl alcohol (*n*-BuOH), and water. Antioxidant activity (DPPH assay) was done along with thin layer chromatography (TLC) for the selection of fractions for further isolation, which led to the selection of the *n*-BuOH fraction for further study. For the isolation of compounds, the BuOH fraction was chromatographed on a Sephadex column and eluted with 20 to 100% methanol, which gave eleven fractions labeled 1-11. Fractions 2–7, and 10 were further chromatographed on Sephadex LH-20 (25–100 μm) and an ODS column (filled with a octadecylsilyl group packing) in order to get pure compounds labeled **1–15**. For the elucidation of the structures of the isolated compounds, samples were dissolved in MeOH-d_4_ (CD_3_OD) and then analyzed by H-NMR and ^13^C-NMR. The mass spectra of the compounds was detected by using FAB in negative mode for correct identification.

### 4.4. Development of a Fingerprint Pattern of Nigella sativa by HPLC-UV along with Quantitative and Qualitative Analysis

A sample (0.36 g) of the powdered aerial parts of *N. sativa* was weighed and extracted with 25 mL of 70% alcohol at room temperature for 90 min. The extracted solution was centrifuged at 15,000 rpm for 5 min, filtered through a 0.22 μm membrane filter, and kept at 4 °C until analysis. Peak analysis was done with standard compounds and identification by their UV spectra and retention time using HPLC chromatography with UV detection. A RP-C_18_ Kromasil column (250 × 4.6 mm, 5 μm) was used with a 1 mL/min flow rate of a mobile phase gradient of water with 0.1% FA in water (solvent A) and acetonitrile and methanol in a 1:1 ratio (solvent B): 0 min, 80% A; 10 min, 78% A; 30 min, 78% A; 50 min, 75%A; 80 min, 70% A, 100 min, 65% A, and 110 min, 10%A. The column was equilibrated with 90% A for 10 min before next injection with and the detection wavelength was 365 nm. The injected volume was 20 μL. The column temperature was kept at 40 °C.

### 4.5. Bioassays

#### 4.5.1. Antioxidant assays

##### 1-Diphenyl-2-picryl-hydrazyl (DPPH) Assay

The DPPH activity of the isolated compounds and extracts were evaluated as described previously [34]. Twenty μL of samples having different concentrations of the extract (100, 500, 250, and 125 µg/mL) and isolated compounds (100, 75, 50, and 25 μM) keeping the total volume of solution at 180 μL (0.1 mM) solution, was added in 96 well plates and incubated for 30 min at 37 °C. The observation was measured at 517nm. Each observation was done in triplicate. Ascorbic acid and Trolox were used as positive controls, while methanol was used as a negative control. Inhibition % was calculated. Data were presented as the mean ± S.D.

##### 2′-Azino-bis (3-ethylbenzothiazoline-6-sulphonic acid (ABTS) Assay

ABTS inhibition of the isolated compounds and extracts were evaluated as described previously [34]. Stock solution was prepared by mixing an equal volume of 7.4 mM of ABTS and 2.6 mM of potassium persulfate and kept for 24 hours before diluting with methanol. Later the solution was diluted with methanol till the absorbance at 732 nm came within the range of 0.8–1.2. To 50 µL of sample solutions of different concentration (250, 125, 100, 50 μM) 950 μL of ABTS* was added. Absorbance was measured at 732 nm by a spectrophotometer. Ascorbic acid and Trolox, and methanol were used as a positive and negative controls, respectively. All observations were done in triplicate. Percentage inhibition was calculated from the observations. Data were presented as mean ± S.D.

#### 4.5.2. Anti-diabetic Assays

##### α-Glucosidase Assay

Enzyme inhibition was investigated spectrophotometrically in a 96 well plate. A total of 60 μL of reaction mixture containing 20 μL of 100 mM potassium phosphate buffer (pH 6.8), and 20 μL of 2.5 mM of *p*-nitrophenyl-α-d-glucopyranoside in the buffer and 20 μL samples of different concentrations (100, 50, 25, and 12.5 μM) dissolved in 10% DMSO were added to each well. Subsequently, 20 μL of 10 μM phosphate buffer (pH 6.8) containing 0.2 U/mL α-glucosidase was added. The plate was incubated for 15 min at 37 °C the reaction was terminated by adding 80 μL of 0.2 mM sodium carbonate solution and the absorbance was measured at 405 nm. Acarbose (100, 50, 25, and 12.5 μM) was used as a positive standard. The control plate contained the same volume of buffer solution instead of the sample solution. The percentage inhibition was calculated using the following equation [(Ac−AsAc)]×100%, where $A_{c}$ is the absorbance of control while $A_{s}$ is the absorbance of the sample. Data were presented as a mean ± S.D [35].

##### Protein Tyrosine Phosphatase 1B (PTP1B) Inhibitory Assay

The inhibitory activity of the isolated compounds against human recombinant PTP1B was evaluated using *p*-nitrophenyl phosphate (pNPP) as a substrate. Firstly, 2 mM pNPP and PTP1B were mixed with 50 mM citrate buffer of pH 6 and 0.1 mM NaCl, 1 mM EDTA, and 1 mM DTT with or without sample keeping the final volume of the solution at 110 μL and placing them in a 96 wells plate. Samples with different concentrations (100, 50, 25, and 12.5 μM) were previously dissolved in 10% DMSO. The plate was first pre-incubated for 10 min at 37 °C, and then 50 μL of pNPP in buffer was added. The plate was again incubated for 30 min at 37 °C. After that, 10 μL of 10M NaOH was added dropwise to stop the reaction. The amount of *p*-nitrophenyl produced after the enzymatic dephosphorylation was calculated by measuring the absorbance at 405 nm. The non-enzymatic hydrolysis of 2 mM pNNP was corrected by measuring the increase in absorbance at 405 nm, which was obtained in the absence of the PTP1B enzyme. The inhibition percentage was calculated with the formula [(Ac−AsAc)] × 100%, where $A_{c}$ is the absorbance of control while *A_s_* is the absorbance of the sample. In this experiment, Ursolic acid with different concentrations (10, 7.5, 5, and 0.25 μM) was added as a positive control. Data were presented as mean ± S.D [35].

## Figures and Tables

**Table 1:** IC_50_ values of the bioassays of the compounds isolated from *Nigella sativa.*

Compound Number	DPPH Assay	ABTS Assay	α-Glucosidase Assay	PTP1B Assay
**1**	Nd	Nd	217.5 ± 2.6	Nd
**2**	Nd	Nd	256.7 ± 3.7	Nd
**3**	Nd	Nd	Nd	Nd
**4**	Nd	Nd	254.2 ± 4.5	Nd
**5**	71.0 ± 0.5	139.2 ± 0.5	335.3 ± 0.2	Nd
**6**	32.7 ± 0.1	95.18 ± 0.9	257.8 ± 0.8	Nd
**7**	Nd	Nd	276.2 ± 2.1	Nd
**8**	35.5 ± 0.5	98.8 ± 0.5	274.1 ± 0.3	Nd
**9**	197.8 ± 2.7	247 ± 2.7	360.0 ± 0.3	Nd
**10**	Nd	Nd	214.5 ± 0.0	Nd
**11**	39.6 ± 0.5	129.0 ± 0.5	331.9 ± 1.6	Nd
**12**	Nd	Nd	Nt	91.3 ± 2.5
**13**	Nd	Nd	Nt	Nd
**14**	Nd	Nd	Nt	Nd
**15**	Nd	Nd	Nt	Nd
Ascorbic acid	51.7 ± 0.1	82.9 ± 2	Nt	Nt
Trolox	59.4 ± 0.9	96.2 ± 1.5	Nt	Nt
Acarbose	Nt	Nt	127.9 ± 2.0	Nt
Ursolic acid	Nt	Nt	Nt	0.8 ± 1.4

The values are presented as the mean of three determination ± standard deviation. Nt (not tested), Nd (not detected). Ascorbic acid and Trolox were used as a positive control in the DPPH and ABTS assays. Acarbose and ursolic acid were used as positive controls in the α-glucosidase assay and PTP1B assay, respectively.

## Supplementary Materials

Supplementary File 1
