# Supplementary material for: A New Oleanane Type Saponin from the Aerial Parts of Nigella sativa with Anti-Oxidant and Anti-Diabetic Potential
Source: Molecules. 2020 May 6;25(9):2171. doi: 10.3390/molecules25092171 (PMC7248923; doi:10.3390/molecules25092171)
Supplement: Supplementary File 1 [file molecules-25-02171-s001.pdf]

HNMR Data

1: 3-O-〔β-D-xylopyranosyl-(1-3)-α-L-rhamnopyrnaosyl-(1-2)-α-L-arabinopyranosyl〕-28-O-〔α-L-rhamnopyrnaosyl-(1-4)-β-D-glucopyranosyl-(1-6)-β-D- glucopyranosyl〕-hederagenin: ^{1} **HNMR (CD_3OD, 600MHz):** δ 5.35 (1H, d, J= 8.22, H-1′′′′′), 5.31 (1H, d, J= 8.16, H-1′′′′′′), δ 5.25 (1H, t, H-12), 5.22 (1H, s, H-1′′), 5.22 (1H, s, H-1′′′′), 4.55 (1H, d, J= 5.28 H-1′′′), 4.51 (1H, d, J=5.64, H-1′), 3.1-4.1 (sugar moieties overlapped), 0.70 (3H, s, H-24) 0.79 (3H, s, H-26), 0.90 (3H, s, H-29), 0.94 (3H, s, H-30), 0.97 (3H, s, H-25), 1.16 (s, 3H each, H-27) 1.23 (m, 6H, 2CH3, H-6′′ & H-6′′′′). ^{13} **CNMR (CD_3OD, 600MHz)** δ 12.41 (C-24), 15.13 (C-25), 16.46 (C-6′′′′), 16.59 (C-6′′), 17.43 (C-6), 22.63 (C-30), 22.67 (C-16), 23.15(C-11), 24.93(C-27), 25.01(C-2), 27.52(C-15), 30.12(C-20), 31.01(C-7 & 22), 31.94(C-29). 33.48 (C-21), 36.23 (C-10), 38.2 (C-1), 38.38(C-8), 41.12(C-18), 41.61(C-14), 42.58(C-4), 45.82(C-17), 46.3 (C-19), 46.65 (C-5), 48.1 (C-9), 63.18 (C-23), 76.58 (C-3), 94.35 (C-1′′′′′), 101.1(C-1′′′′),101.3 (C-1′′)102.95 (C-1′′′), 103.22 (H-1′′′′′′), 105.15 (C-1′), 122.39 (C-12), 143.50 (C-13), 176.68 (C-28) and other signals are similar according to literature.

2: Compound 2: Flaccidoside III (first time from genus nigella): ^{1} **HNMR (CD_3OD, 600MHz)** δ 5.33 (1H, d, J= 7.38, H-1′′′′′), 5.25 (1H, t, H-12), 5.23 (1H, s, H-1′′), 5.22 (1H, s, H-1′′′′), 4.51 (1H, d, J=5.58, H-1′), δ 4.49 (1H, d, J= 7.38, H-1′′′′′), δ 4.41 (1H, d, J= 7.38, H-1′′′), 3.3-4.1 (sugar moieties overlapped), 0.70 (3H, s, H-24) 0.80 (3H, s, H-26), 0.91 (3H, s, H-29), 0.94 (3H, s, H-30), 0.98 (3H, s, H-25), 1.16 (s, 3H each, H-27) 1.23 (m, 6H, 2CH3, H-6′′ & H-6′′′′). ^{13} **CNMR (CD_3OD, 600MHz)** δ 176.74 (C-28), 143.51 (C-13), 122.68 (C-12), 105.15 (C-1′), 103.3 (C-1′′′), 101.52(C-1′′), 99.93 (C-1′′′′), 94.35 (C-1′′′′′), 76.58 (C-3), 65.60 (C-6′′′), 64.10 (C-6′′′′), 63.18 (C-23), 60.47 (C-6′′′′′), 48.1 (C-9), 46.65 (C-5), 46.3 (C-19), 45.82 (C-17), 42.58 (C-4), 41.61(C-14), 41.11 (C-18), 39.27 (C-8), 38.33 (C-1), 36.24 (C-10), 33.52 (C-21), 32.09 (C-29), 31.87 (C-7 & 22), 30.14 (C-20), 27.53 (C-15), 25.20 (C-2), 24.97(C-27), 23.17(C-11), 22.64 (C-16), 22.64 (C-30), 17.44 (C-6), 16.62 (C-6′′), 15.17 (C-25), 12.44 (C-24), and other signals are similar according to literature.

3: Catechol: ^{1} **HNMR (CD_3OD, 600MHz)** δ 8.18(s, 2H, H-3 & 6), 8.11 (s, 2H, H-4 & 5)^{13} **CNMR (CD3OD, 600MHz)** δ 152.25 (C-1 & 2), 139.79 (C-3 & 6)**.**

4: Quercetin-3-gentiobiosides: ^{1} **HNMR (CD_3OD, 600MHz)** δ 7.77 (1H, dd, J=2.16, 8.58, H-6′), 7.65 (1H, d, J=2.22, H-2′), 7.35 (1H, d, J=8.64, H-5′), 6.3 (1H, s, H-8), 6.1 (1H, s, H-6), 5.03 (1H, d, J=7.26, H-1′′), 4.72 (1H, d, J=7.74, H-1′′′) 3.2-3.9(disaccharide moieties
overlapped)**.** ^{13} **CNMR (CD_3OD, 600MHz)** 159.0 (C-2), 135.0 (C-3), 179.6 (C-4), 163.0 (C-5), 99.8 (C-6), 166.0 (C-7), 94.7 (C-8), 158.4 (C-9), 105.1 (C-10), 122.8 (C-1′), 116.2 (C-2′), 146.1 (C-3′), 149.8 (C-4′), 117.4 (C-5′), 123.1 (C-6′), 104.5 (C-1′′), 75.6 (C-2′′), 78.2 (C-3′′), 71.2 (C-4′′), 77.7 (C-5′′), 66.2 (C-6′′).

5: Magnoflorine: **^{1}HNMR (CD_3OD, 600MHz)** 6.61 (1H, d, J=7.86, H-8), 6.38 (1H, d, J=7.8, H-9), 6.34 (1H, s, H-3), 3.80 (3H, s, OMe-2), 3.71 (3H, s, OMe-10), 3.60 (2H, m, H-5), 3.14 (3H, s, N-CH3), 3.02 (2H, m, H-4), 2.65 (3H, s, N-CH3).**^{13}CNMR (CD_3OD, 600MHz)**

151.51 (C-2), 150.2 (C-10), 149.34 (C-11), 148.40 (C-1), 124.6 (C-7a), 122 (C-8), 119.59 (C-3a), 115.5 (11-a), 114.3 (1-a), 109.13 (C-9), 107.94 (C-3), 69.44 (C-6a), 60.74 (C-5), 54.89 (OMe-C10), 54.59 (OMe C11), 52.43, 42.122 (N^+Me), 30.15 (C-7), 23.18 (C-4).

6: Nigelflavonoside B: ^{1}HNMR (CD_3OD, 600MHz) δ 7.7 (1H, d, J=2.16, H-6′), 7.55 (1H, dd, J=2.16, 8.46, H-2′), 6.91 (1H, d, J=8.46, H-3′), 6.36 (1H, s, H-8), 6.17 (1H, s, H-6), 5.24 (1H, d, J=7.26, H-1″), 4.73 (1H, d, J=7.74, H-1‴), 4.67 (1H, d, J=7.68, H-1⁗), 4.50 (1H,s, H-1⁗′), 3.2-3.9 (disaccharide moieties overlapped), 1.10 (3H, d, H-6⁗′). **^{13}CNMR (CD_3OD, 600MHz)** 177.9 (C-4), 164.38 (C-7), 161.6 (C-5), 157.03 (C-9 & 2), 148.3 (C-4′), 144.30 (C-3′), 133.4 (C-3), 121.84 (C-1′ & 6′), 116.43 (C-2′), 114.84 (C-5′), 105.01 (C-10), 104.25 (C-1⁗), 103.61 (C-1‴), 100.67 (1⁗′), 99.59 (C-6), 98.47 (C-1″), 83.83 (C-2″), 82.27 (C-2‴), 77.39 (C- 5⁗), 76.19 (C-3⁗), 75.93 (C-3″), 75.65 (C-5″), 74.91 (5‴), 73.63(C-3‴), 72.49 (C-6″), 70.74 (C-4⁗′), 70.64 (C-3⁗′), 69.59 (C-2⁗′), 69.16 (C-4″), 60.82 (C-6⁗), 60.43 (C-6‴), 16.45 (C-6⁗′).

Nigelloside: ^{1}HNMR (CD_3OD, 600MHz) δ 8.05 (2H, d, J=8.82, H-2′ & 6′), 6.94 (2H, d, J=8.82, H-3′ & 5′), 6.36 (1H, s, H-8), 6.17 (1H, s, H-6), 5.39 (1H, d, J=7.5, H-1″), 4.73 (1H, d, J=7.68, H-1‴), 4.67 (1H, d, J=7.68, H-1⁗), 4.50 (1H,s, H-1⁗′), 3.2-3.9(disaccharide moieties overlapped), 1.13 (3H, d, H-6⁗′). **^{13}CNMR (CD_3OD, 600MHz)** 178.1 (C-4), 164.43 (C-7), 161.63 (C-5), 159.96 (C-4′), 157.06 (C-2,9), 133.2 (C-3), 131.84 (C-2′ & 6′), 121.43 (C-1′), 114.89 (C-3′, 5′), 104.33 (C-10), 103.39 (C-1″), 100.72 (1⁗), 99.40 (C-1⁗′), 98.47 (C-6), 83.83 (C-2″), 82.27 (C-2‴), 77.39 (C- 5⁗), 76.19 (3⁗), 75.93 (C-3″), 75.65 (C-5″), 74.91 (5‴), 73.59(C-3‴), 72.49 (C-6″), 70.74 (C-4⁗′), 70.64 (C-3⁗′), 69.59 (C-2⁗′), 69.29 (C-4″), 68.27 (C-4⁗), 66.57 (C-6″), 60.90 (C-6⁗), 60.43 (C-6‴), 16.45 (C-6⁗′).

8: Quercetin sphorotrioside: ^{1}HNMR (CD_3OD, 600MHz) δ 7.75 (1H, d, J=2.16, H-6′), 7.55 (1H, dd, J=2.16, 8.46, H-2′), 6.91 (1H, d, J=8.46, H-3′), 6.35 (1H, s, H-8), 6.16 (1H, s, H-6), 5.29 (1H, d, J=7.14, H-1″), 4.73 (1H, d, J=7.68, H-1‴), 4.67 (1H, d, J=7.38, H-1⁗), 3.2-3.9(disaccharide moieties overlapped), 1.10 (3H, d, H-6⁗′). **^{13}CNMR (CD_3OD, 600MHz)** 177.9 (C-4), 164.38 (C-7), 161.67 (C-5), 157.03 (C-9 & 2), 148.3 (C-4′), 144.30 (C-3′), 133.4 (C-3), 121.84 (C-1′ & 6′), 116.36 (C-2′), 114.83 (C-5′), 105.07 (C-1⁗), 104.35 (C-1‴), 103.659 (C-10), 99.61 (C-6), 98.47 (C-1″), 83.83 (C-2″), 82.27 (C-2‴), 77.39 (C-5‴), 76.95 (C-5⁗)76.19 (C-5″), 75.93 (C-3″), 75.65 (C-5″), 74.91 (3″),74.78 (C-3⁗), 73.63(C-2⁗), 69.59 (C-4″), 68.83 (C-4⁗),68.26 (C-6″) 60.93 (C-6⁗), 60.39 (C-6‴).

9: kaempferol-3, 7-diglucoside:^{1}HNMR (CD_3OD, 600MHz)

δ 8.08 (2H, d, J=8.88, H-2′ & 6′), 6.90 (2H, d, J=8.76, H-3′ & 5′), 6.38 (1H, s, H-8), 6.19 (1H, s, H-6), 5.33 (1H, d, J=7.62, H-1″), 4.74 (1H, d, J=7.68, H-1‴), 3.3-3.8(sugar moeities).

^{13} **CNMR (CD_3OD, 600MHz)** 178.38 (C-4), 164.82 (C-7), 160.14 (C-5), 157.22 (C-4′), 157.08 (C-2,9), 133.48 (C-3), 130.94 (C-2′ & 6′), 121.27 (C-1′), 114.84 (C-3′, 5′), 104.22 (C-10), 103.39 (C-1‴), 100.08 (1″), 98.50 (C-6), 93.33 (C-8), 77.4 (C-3″,5″,3‴, 5‴) 74.06 & 73.42 (C-2‴ &2″), 69.89 (C-4″), 69.7(C-4‴), 61.1 (C-6″, 6‴).

10: kaempferol 3-O-rutinoside: ^{1} **HNMR (CD_3OD, 600MHz)** δ 8.05 (2H, d, J=8.76, H-2′ & 6′), 6.87 (2H, d, J=8.76, H-3′ & 5′), 6.38 (1H, s, H-8), 6.19 (1H, s, H-6), 5.12 (1H, d, J=7.44, H-1″), 4.51 (1H, s, H-1‴), 3.3-3.8(sugar moeities), 1.12 (3H, d, J=6.20, H-6‴) ^{13} **CNMR (CD_3OD, 600MHz)** 177.94 (C-4), 164.64 (C-7), 161.53 (C-5), 160.04 (C-4′), 157.96 (C-9), 157.08 (C-2), 134.10 (C-3), 130.94 (C-2′ & 6′), 121.30 (C-1′), 114.71 (C-3′, 5′), 104.22 (C-10), 103.22 (C-1″), 101.08 (1‴), 98.50 (C-6), 93.33 (C-8), 76.72 (C-3″), 75.77 (C-5″), 74.31 (C-2″), 72.48 (C-4″), 70.068(C-4‴), 70.61 (C-2‴), 69.99 (C-3‴), 68.30 (C-5‴), 67.17 (C-6″), 16.50 (C-6‴).

11: Rutin: ^{1} **HNMR (DMSO-d_6, 600MHz)** δ 7.5 (2H, s, H-2′ & 6′), 6.8 (1H, s, H-5′), 6.36 (1H, s, H-8), 6.17 (1H, s, H-6), 5.31 (1H, s, H-1″), 4.36 (1H, s, H-1‴), 3.2-3.9(disaccharide moieties overlapped), 0.97 (3H, H-6⁗). ^{13} **CNMR (DMSO-d_6 600MHz)** 177.67 (C-4), 164.86 (C-7), 161.63 (C-5), 156.96 (C-9 & 2), 148.95 (C-4′), 145.22 (C-3′), 133.69 (C-3), 122.03 (C-1′), 121.53 (c-6′), 116.43 (C-5′), 114.84 (C-2′), 104.21 (C-10), 101.72 (1‴), 101.17 (C-1‴), 99.29 (C-6), 94.15 (C-8),76.90 (C-3″), 76.32 (C-5″), 74.50 (C-2″), 72.28 (C-4‴), 70.99 (C-4″, 2‴), 70.41 (C-3‴), 68.65 (C-5‴), 67.39 (C-6″), 18.17 (C-6‴).

12: 3-O-[α-L-rhamnopyranosyl-(1→2)-α-L-arabinopyranpsyl]-hederagenin: ^{1} **HNMR**
(CD_3OD, 600MHz) δ 5.15 (1H, t, H-12), 5.14 (1H, s, H-1″), 4.55 (1H, d, J= 5.16, H-1‴), 3.2-3.9 (sugar moieties), 0.69 (3H, s, H-24) 0.82 (3H, s, H-26), 0.93 (3H, s, H-29), 0.96 (3H, s, H-30), 0.99 (3H, s, H-25), 1.18 (s, 3H each, H-27) 1.23 (3H, CH3, H-6‴). ^{13} **CNMR (CD_3OD, 600MHz)** δ 178.88 (C-28), 142.93 (C-13), 122.56 (C-12), 102.87 (C-1″), 100.47 (C-1′), 80.84 (C-3),75.25 (C-2″) 72.51 (C-3′), 72.23(C-4′), 71.14 (C-3′), 70.73 (C-2′), 70.60 (C-4″), 68.75 (C-5′, & 5″), 63.18 (C-23), 52.19 (C-9), 47.45 (C-5), 47.59 (C-19), 47.51 (C-17), 42.77 (C-4), 42.52 (C-14), 41.84 (C-18), 39.29 (C-8), 38.23 (C-1), 36.18 (C-10), 32.18 (C-21), 31.87 (C-29), 30.76 (C-7 & 22), 26.90 (C-20), 25.58 (C-15), 25.09(C-2), 23.79 (C-27), 23.13 (C-11 & 30), 17.39 (C-16), 16.55 (C-6), 16.38 (C-6′), 15.58 (C-26) , 14.94 (C-25), 12.32 (C-24).

13:3b,23,28-trihydroxyolean-12-ene 3-O-a-L-arabinopyranoside (1__4)-a-L-rhamnopyranosyl (1__ 4)-b-D-glucopyranoside (new compound): ^{1} **HNMR (C_5D_5N, 600MHz)** δ 5.22 (1H, s, H-12), 3.2-3.9 (sugar moieties), 3.82 (1H, H-3), 3.50 (2H, H-23), 3.34 (2H, H-28), 0.70 (3H, s, H-24) 0.87 (3H, s, H-26), 0.92 (3H, s, H-29), 0.97 (6H, H-25 & 30), 1.07 (3H, s, 27), 1.26 (3H, d, J=6.18, H-6″). ^{13} **CNMR (C_5D_5N, 600MHz)** 144.70 (C-13), 121.70 (C-12), 105.08 (C-1′), 103.21 (C-1‴), 99.99 (C-1″), 80.99 (C-3′), 80.68 (C-3 & 3‴), 76.20 (C-5′), 74.79 (C-4′), 73.81 (C-2″), 72.72 (C-4″), 71.36 (C-3″), 70.25 (C-2‴), 69.66 (C-2′), 68.54 (C-5″), 68.20 (C-5‴), 65.59 (C-6′), 63.24 (C-23), 52.17 (C-17), 46.78 (C-5),

46.12 (C-19), 42.58 (C-4), 42.17 (C-14), 42.16 (C-18), 41.29 (C-1 & 21), 39.19 (C-8), 38.30 (C-5‴), 36.26 (C-10), 32.41 (C-29), 32.12(C-7), 32.12 (C-22 & 7), 30.65 (C-20), 25.20 (C-15), 27.36 (C-2), 25.27 (C-27), 23.98 (C-30 & 11), 23.8 (C-16), 17.50 (C-6), 16.80 (C-26), 16.66 (C-6″), 15.12 (C-25), 12.46 (C-24).

14: 3-O-[α-L-rhamnopyranosyl-(1→2)-α-L-arabinopyranpsyl]-28-o-β-d-glucopyranosyl hederagenin: **^{1} HNMR (C_5D_5N, 600MHz)** δ 6.33 (1H, s, H-12), 5.35 (1H, d, J= 7.38 H-1‴), 5.09 (1H, d, J= 6.42, H-1″), 4.92 (1H, s, H-1′) 3.2-3.9 (sugar moieties), 0.93 (3H, s, H-24) 0.96 (3H, s, H-26), 1.02 (3H, s, H-29), 1.15 (3H, s, 27), 1.26 (6H, s, H-30 &25), 1.58 (3H, CH3, H-6‴). **^{13} CNMR (C_5D_5N, 600MHz)** δ 178.88 (C-28), 142.93 (C-13), 122.56 (C-12), 106.09 (C-1″), 103.24 (C-1′), 99.92 (C-1‴), 81.61 (C-3), 79.72 (C-3‴), 79.70 (C-5‴), 76.91 (C-2″) 74.8 (C-3‴) 74.18 (C-4′ &2‴), 73.84 (C-3′), 72.4 (C-2′), 71.52 (C-3‴), 69.7 (C-4″), 68.28 (C-5′), (C-4″), 65.94 (C-5″), 64.74 (C-23), 62.63 (C-6‴), 52.19 (C-9), 47.45 (C-5), 47.59 (C-19), 47.51 (C-17), 42.77 (C-4), 42.52 (C-14), 40.84 (C-18), 39.62 (C-8), 38.30 (C-1), 37.63 (C-10), 32.00 (C-21), 31.49 (C-29), 29.65 (C-7), 28.56 (C-22), 26.90 (C-20), 25.58 (C-15), 24. 74 (C-2), 23.79 (C-27), 22.65 (C-11 & 30), 17.39 (C-16), 16.55 (C-6), 16.38 (C-6′), 15.58 (C-26), 14.94 (C-25), 12.32 (C-24).

15: α-hederin: **^{1} HNMR (C_5D_5N, 600MHz)** δ 6.52 (1H, s, H-12), 5.71 (1H, s, H-1″), 5.41 (1H, d, J= 6.12, H-1″), 3.2-3.9 (sugar moieties), 1.21 (3H, s, H-24) 1.24 (3H, s, H-26), 1.30 (3H, s, H-29), 1.36 (3H, s, 27), 1.52 (6H, s, H-30 &25), 1.83 (3H, CH3, H-6″). **^{13} CNMR (C_5D_5N, 600MHz)** δ 180.1 (C-28), 145.46 (C-13), 122.15 (C-12), 104.42 (C-1′), 101.24 (C-1″), 81.61 (C-3), 75.89 (C-2′), 74.75 (C-3′), 74.17(C-4″), 72.63 (C-3″), 72.41 (C-2″), 69.77 (C-5′), 69.40 (C-5″), 65.77 (C-6′), 64.11 (C-23), 48.28 (C-9), 47.81 (C-4), 46.94 (C-17), 43.59 (C-19 & 5), 42.28 (C-14), 41.07 (C-18), 39.80 (C-8), 39.06 (C-1), 36.99 (C-10), 34.52 (C-21), 33.44 (C-29), 32.96 (C-7 & 22), 31.10 (C-20), 28.59 (C-15), 26.36 (C-27), 26.30 (C-2), 24.03 (C-30), 23.92 (C-11 & 16), 18.62 (C-6), 17.67 (C-26), 16.18 (C-25), 14.07 (C-24).

Table 1: NMR data of compound 13

C#No	C NMR	H NMR	C NMR of sugar	HNMR sugar
1	41.29		**Glucose (C′)**	
2	27.36		105.08	4.50 (1H, d, J=7.38, H-1)
3	80.68	3.82 (1H)	69.66	3.4-3.5 (1H, m, H-2)
4	42.58		80.99	3.60 (1H, dd, J= 4.36 & 11.20, H-3)
5	46.78		74.79	3.70 (1H, d, J=12.66 H-2)
6	17.50		76.20	3.34 (1H, m, H-5)
7	32.12		65.59	3.92 (2H, m, H-6)
8	39.19			

9	47.66		**Rhamnose (C′′)**	
10	36.26		99.99	5.22 (1H, s, H-1)
11	23.8		73.81	3.31 (1H, m, H-2)
12	121.70	5.22 (1H, s)	71.36	3.53 (1H, t, H-4)
13	144.70		72.72	3.70 (1H, d, J= 8.11, H-4)
14	42.17		68.54	3.94 (1H, m, H-5)
15	25.20		16.66	1.26 (3H, d, J=6.18, H-6)
16	23.8			
17	52.17		**Xylose (C′′′)**	
18	42.16		103.21	4.53 (1H, d, J= 5.16, H-1)
19	46.12		70.25	4.09 (1H, s, H-3)
120	30.65		80.67	3.83 (1H, m, H-3)
21	41.29		68.20	3.77 (1H, brs, H-4)
22	32.12		38.30	1.62 (2H, m, H-5)

23	63.24	3.50 (2H)		
24	12.46	0.70 (3H, s)		
25	15.12	0.97 (3H, s)		
26	16.80	0.87 (3H, s)		
27	25.27	1.07 (3H, s)		
28	64.00	3.34 (2H)		
29	32.41	0.92 (3H, s)		
30	23.98	0.97 (3H, s)		

Table 2: IC50 value of bioassays of isolated compounds from *Nigella sativa*

Sr#No	IC50 (µM)				
	**Compounds name**	**DPPH assay**	**ABTS assay**	**α-glucosidase assay**	**PTP1B assay**
	3-O-[β-D-xylopyranosyl-(1-3)-α-L-rhamnopyrnaosyl-(1-2)-α-L-				

1.	arabinopyranosyl]- 28-O-[α-L-rhamnopyrnaosyl-(1-4)-β-D-glucopyranosyl-(1-6)-β-D-glucopyranosyl]-hederagenin	Nd	Nd	217.51 ± 2.63	Nd
2.	Flaccidoside III	Nd	Nd	256.71 ± 3.79	Nd
3.	Catechol	Nd	Nd	Nd	Nd
4.	quercetin-3-gentiobiosides	Nd	Nd	254.29 ± 4.51	Nd

5.	Magnoflorine	71.03 ± 0.5	139.2 ± 0.5	335.38 ± 0.2	Nd
6.	Nigelflavonoside B	32.76 ± 0.12	95.18 ± 0.9	257.86 ± 0.88	Nd
7.	Nigelloside	Nd	Nd	276.23 ± 2.1	Nd
8.	Quercetin sphorotrioside	35.5 ± 0.5	98.87 ± 0.5	274.1 ± 0.32	Nd
9.	kaempferol-3, 7-diglucoside	197.8 ± 2.7	247 ± 2.79	360.0 ± 0.39	Nd
10.	kaempferol 3-O-rutinoside	Nd	Nd	214.53 ± 0.09	Nd
11.	Rutin	39.67 ± 0.5	129.00 ± 0.55	331.93 ± 1.69	Nd
12.	3-O-[α-L-rhamnopyranosyl-	Nd	Nd	Nt	91.30 ± 2.5

	(1→2)-α-L-arabinopyranpsyl]-<br><br>hederagenin				
13.	3b, 23, 28-trihydroxyolean-12-ene-3-O-a-L-arabinopyranoside (1__4)-a-L-rhamnopyranosyl (1__ 4)-b-D-glucopyranoside	Nd	Nd	Nt	Nd

14.	3-O-〔α-L-<br>rhamnopyranosyl-<br>(1→2)-α-L-<br>arabinopyranpsyl〕-<br>28-<br>o-β-d-<br>glucopyranosyl<br>hederagenin	Nd	Nd	Nt	Nd
15.	α-hederin	Nd	Nd	Nt	Nd
16.	Ascorbic acid	51.73 ± 0.1	Nt	Nt	Nt
17.	Trolox	59.48 ± 0.9	Nt	Nt	Nt
18.	Allopurinol	Nt	92.54 ± 2.3	Nt	Nt
19.	Acarbose	Nt	Nt	127.93 ± 2.02	Nt

20.	Ursolic acid	Nt	Nt	Nt	0.85 ± 1.4

Nt: not tested, Nd: not detected
